# Proteasome Inhibition Suppresses KIT-Independent Gastrointestinal Stromal Tumors Via Targeting Hippo/YAP/Cyclin D1 Signaling

**DOI:** 10.3389/fphar.2021.686874

**Published:** 2021-05-06

**Authors:** Ting Chen, Nan Ni, Li Yuan, Liangliang Xu, Nacef Bahri, Boshu Sun, Yuehong Wu, Wen-Bin Ou

**Affiliations:** ^1^ Zhejiang Provincial Key Laboratory of Silkworm Bioreactor and Biomedicine, College of Life Sciences and Medicine, Zhejiang Sci-Tech University, Hangzhou, China; ^2^ Department of Pathology, Brigham and Women’s Hospital and Harvard Medical School, Boston, MA, United States

**Keywords:** cyclin D1, hippo pathway, bortezomib, KIT-independent GIST, YAP/TAZ

## Abstract

**Purpose:** Gastrointestinal stromal tumors (GISTs) are the most common malignant tumor of mesenchymal origin of the digestive tract. A yet more challenging resistance mechanism involves transition from oncogenic KIT to a new imatinib-insensitive oncogenic driver, heralded by loss of KIT expression. Our recent studies have shown that inhibition of cyclin D1 and Hippo signaling, which are overexpressed in KIT-independent GIST, is accompanied by anti-proliferative and apoptosis-promoting effects. PRKCQ, JUN, and the Hippo/YAP pathway coordinately regulate GIST cyclin D1 expression. Thus, targeting of these pathways could be effective therapeutically for these now untreatable tumors.

**Methods:** Targeting cyclin D1 expression of small molecular drugs was screened by a cell monolayer growth and western blotting. The biologic mechanisms of bortezomib to KIT-independent GISTs were assessed by immunoblotting, qRT-PCR, cell viability, colony growth, cell cycle analysis, apoptosis, migration and invasiveness.

**Results:** In the initial small molecular inhibitor screening in KIT-independent GIST62, we found that bortezomib-mediated inhibition of the ubiquitin-proteasome machinery showed anti-proliferative effects of KIT-independent GIST cells via downregulation of cyclin D1 and induction of p53 and p21. Treatment with proteasome inhibitor, bortezomib, led to downregulation of cyclin D1 and YAP/TAZ and an increase in the cleaved PARP expression in three KIT-independent GIST cell lines (GIST48B, GIST54, and GIST226). Additionally, it induced p53 and p21 expression in GIST48B and GIST54, increased apoptosis, and led to cell cycle G1/G2-phase arrest, decreased cell viability, colony formation, as well as migration and invasiveness in all GIST cell lines.

**Conclusion:** Although our findings are early proof-of-principle, there are signs of a potential effective treatment for KIT-independent GISTs, the data highlight that targeting of cyclin D1 and Hippo/YAP by bortezomib warrants evaluation as a novel therapeutic strategy in KIT-independent GISTs.

## Introduction

Most gastrointestinal stromal tumors (GISTs) are driven by activating oncogenic mutations of receptor tyrosine kinase (RTK) KIT/PDGFRA, which provide a compelling therapeutic target, as evidenced by the clinical successes of KIT/PDGFRA-inhibition by small molecule therapeutics. More than 85% of GIST patients treated with the small molecule drugs such as first line-imatinib, second line-sunitinib, or third line-regorafenib, achieve disease stabilization. But, a substantial proportion of patients develop resistance to these inhibitors over time (Corless et al., 2004; Dematteo et al., 2009; Chen et al., 2017). The majority of patients with advanced GIST ultimately become resistant to tyrosine kinase inhibitor (TKI) due to secondary KIT or PDGFRA mutations (Gebreyohannes et al., 2019; Huang et al., 2020). Recent studies found that the use of avapritinib (BLU285) to target these KIT or PDGFRA mutations showed significantly better antitumor effects than imatinib or regorafenib (Gebreyohannes et al., 2019), and avapritinib has been approved by the FDA for the treatment of inoperable or metastatic GIST with PDGFRA exon 18 mutation (Dhillon, 2020).

A yet more challenging resistance mechanism found in GIST lesions with KIT mutations as they progress clinically involves the transition from KIT to another novel oncogene, which shows strong resistant to these small molecular inhibitors due to the loss of KIT expression (Fletcher et al., 2003; Debiec-Rychter et al., 2005; Tu et al., 2018; Ou et al., 2019). In our more recent study, we characterized biologic features in KIT-independent, imatinib-resistant GISTs as a step towards identifying actionable drug targets in these poorly understood tumors. We demonstrated that overexpressed cyclin D1 plays an oncogenic role in KIT independent GISTs. Active JUN and Hippo/YAP signaling coordinated and induced cyclin D1 overexpression (Ou et al., 2019). These findings highlight inhibition of cyclin D1 or the Hippo pathway as rational therapeutic strategies for KIT-independent GIST.

Bortezomib is a dipeptide boronic acid inhibitor of the 20S proteasome that is FDA-approved for the treatment of multiple myeloma and mantle cell lymphoma (Adams et al., 1999; Hideshima et al., 2001b; Bross et al., 2004). In multiple myeloma, a key consequence of bortezomib treatment appears to be inhibition of the transcription factor nuclear factor-κB (NF-κB) (Hideshima et al., 2001a; Hideshima et al., 2001b). Early studies found that bortezomib induced internalization and degradation of KIT by binding KIT to ubiquitin-protein ligase CBL which induced primary apoptosis in GIST cells (Dong et al., 2015). Another finding showed that bortezomib rapidly induced apoptosis in GIST cells through H2AX upregulation and loss of KIT protein expression (Bauer et al., 2010). The more recent reports demonstrated that treatment with second-generation inhibitors of the 20S proteasome (delanzomib, carfilzomib and ixazomib) showed strong anti-tumor effects in KIT-dependent GIST cell lines as well as patient-derived tumor xenograft (PDX), including in imatinib-resistant models (Rausch et al., 2020).

In the present study, bortezomib treatment resulted in pro-apoptotic and anti-proliferative effects in KIT independent GIST cell lines through inhibition of cyclin D1 and Hippo/YAP signaling which may be a promising strategy for the future treatment of imatinib-resistant GISTs, and specifically for the as of now untreatable KIT-independent tumors.

## Materials and Methods

A number of the methods described below and KIT-independent GIST cell lines have been described previously (Ou et al., 2019).

### Antibodies and Reagents

Monoclonal mouse antibodies to cyclin D1 (sc-20044) and p53 (sc-126), and monoclonal rabbit antibody to p21 (#29478) were from Santa Cruz Biotechnology (Santa Cruz, CA) and Cell Signaling Technology (Beverly, MA), respectively. Polyclonal antibodies to YAP (#8418) and p-YAP (Ser127, #49118) were from Cell Signaling Technology. PARP (#766606G) and *β*-Actin (A4700) were from Invitrogen Life Technologies (Carlsbad, CA) and Sigma-Aldrich (St, Louis, MO), respectively. 17-allyloamino-17-demethoxygeldanamycin (17-AAG), imatinib, Nutlin-3, and everolimus were obtained from LC Labs (Woburn, MA). Bortezomib was from Selleckchem (Shanghai, China). Inhibitors of MEK1/2 (U0126), PI3-K (LY294002), PLCγ (∆609), and JAK (AG490) were from Calbiochem (SanDiego, California) and the Src family inhibitor, PP1, was from Biomol International L.P. (Plymouth Meeting, PA). All inhibitors were reconstituted in DMSO. Crystal violet and propidium iodide solution were from Sigma-Aldrich. Apoptosis Assays Kit was from Cell Signaling Technology.

### Gastrointestinal Stromal Tumor Cell Lines

GIST48B is isogenic sublines of GIST48, which retains the parental oncogenic KIT genomic mutations but have lost KIT protein-level expression and are therefore KIT-independent. GIST62, GIST54, and GIST226 are KIT-mutant human GIST cell lines, established from KIT-dependent clinically untreated and imatinib-progressing tumors which do not express KIT and are KIT-independent. GIST cell lines were developed in Dr. Jonathan Fletcher’s Laboratory of the Department of Pathology at Brigham and Women’s Hospital. Cells were regularly screened for *mycoplasma* contamination using *Mycoplasma* Stain Assay Kit (Beyotime Biotechnology, Shanghai), and authenticated by SNP array analysis and KIT sequencing prior to these studies. The studies were conducted in accordance with recognized ethical guidelines (United States Common Rule), were approved by Zhejiang Sci-Tech University Institutional Review Boards.

### Cell Culture

GIST62 cell line was maintained in RPMI 1640 medium plus 15% fetal bovine serum (FBS) containing penicillin/streptomycin and L-glutamine. GIST54, GIST226, and GIST48B were maintained in IMDM medium plus 5% or 10% FBS containing penicillin/streptomycin and L-glutamine, respectively. GIST cells were seeded in 6-well plates. Cells were lysed for western blot analysis at 72 h after treatment with bortezomib. Cell culture images were obtained using Spot RT Slider Camera and Spot Software (Version 4.6 for Windows™) and a Nikon Eclipse TE2000-S inverted microscope at 72 h after treatment with different inhibitors.

### Western Blotting

Whole cell lysates from cell lines were prepared using lysis buffer (1% NP-40, 50 mM Tris-HCl pH 8.0, 100 mM sodium fluoride, 30 mM sodium pyrophosphate, 2 mM sodium molybdate, 5 mM EDTA, 2 mM sodium orthovanadate, 10 μg/ml aprotinin, 10 μg/ml leupeptin, 1 mM phenylmethylsulfonyl fluoride, 2 mM Vanadate). Lysates were cleared by centrifugation at 15,000 rpm for 30 min at 4°C, and lysate protein concentrations were determined using a Quick Start™ Bradford Protein Assay (Bio-Rad Laboratories Hercules, CA, United States). Electrophoresis and western blotting were performed as described previously (Rubin et al., 2001), and repeated three independent experiments for each cell line. The hybridization signals were detected by chemiluminescence (ECL, Amersham Pharmacia Biotechnology), captured using a ImageQuant LAS4000 mini chemiluminescence imaging system.

### Ribonucleic Acid Preparation and qRT-Polymerase Chain Reaction

*CCND1*, *YAP*, and *TAZ* RNA expression was evaluated by qRT-PCR in GIST48B and GIST226 cells. Total RNA was prepared using the Trizol reagent (Invitrogen, Carlsbad, CA). Reverse transcription PCR was performed with 0.5 μg RNA, using the PrimeScript™ RT reagent Kit (Takara Bio Inc. Kusatsu, Shiga, Japan). qPCR was performed with TB Green^R^ Premix Ex Taq™ II (Tli RNaseH Plus) (Takara Bio Inc.) in a reaction volume of 25 μL, using an ABI Prism 7500 real-time PCR detection system (Applied Biosystems Inc., Shanghai). Reactions contained 1 μL cDNA, 400 nM of each primer, and 12.5 μL iQ SYBR green supermix. After 3 min at 95°C, each of the 40 PCR cycles consisted of denaturation for 10 seconds at 95°C, hybridization of primers and SYBR green, and DNA synthesis for 1 minute at 60°C. The qRT-PCR assays for *CCND1* were performed using the following primers: sense: 5- CCGTCCATGCGGAAGATC -3’ and anti-sense: 5’-GAA​GAC​CTC​CTC​CTC​GCA​CT-3’ (Thomazy et al., 2002); *YAP* sense: 5’- CCA​CAG​GCA​ATG​CGG​AAT​ATC-3’ and anti-sense: 5’- GGT​GCC​ACT​GTT​AAG​GAA​AGG-3’ (Wang et al., 2017). *TAZ* sense: 5’- GGC​TGG​GAG​ATG​ACC​TTC​AC-3’ and anti-sense: 5’- AGG​CAC​TGG​TGT​GGA​ACT​GAC-3’ (Wang et al., 2017). As a control, *GAPDH* was amplified using the following primers: sense: 5’-GAA​GGT​GAA​GGT​CGG​AGT​CAA​C-3’ and anti-sense: 5’-TGG​AAG​ATG​GTG​ATG​GGA​TTT​C-3’. All primers were obtained from Invitrogen. The comparative C_t_ (cycle threshold) method was used to determine expression differences of *CCND1*, *YAP*, and *TAZ* mRNA in GIST cell lines as compared to the DMSO control after treatment with bortezomib for 24 h. Data (run in triplicate assays) were normalized to *GAPDH*.

### Cell Viability

GIST48B (5,000 cells/well), GIST54 (10,000 cells/well), and GIST226 (15,000 cells/well) were plated in a 96-well flat-bottomed plate (Grenier, Germany) and cultured in IMDM for 24 h before treatment with bortezomib. Viability studies were performed at 3 and 6 days after bortezomib treatment in KIT-independent GIST cell lines. 200 μL MTT (5 mg/ml) for each well was added into GIST cells and incubated for 4 h in the dark. Finally A_490_ was measured by an automatic enzyme-linked immunosorbent assay system AMR-100 (Hangzhou Allsheng Instruments Co.,Ltd., Zhejiang, China). The data were normalized to the DMSO control group. All experimental points were set up in quadruplicate wells and repeated three independent experiments.

### Apoptosis Analysis

Apoptosis was evaluated using the PE Annexin V Apoptosis Detection Kit I (BD Pharmingen). Briefly, GIST48B, GIST54, and GIST226 cells in 6-well plates were treated with bortezomib for 48 h, trypsinized, and washed twice with cold PBS buffer, and then treated with 5 μL of PE Annexin V and 5 μL 7-AAD in 1× binding buffer for 15 min at room temperature (25°C) in the dark. The stained cells were analyzed in a flow cytometer (BD FACS Aria, Special Order System) within 1 h. Flowjo software version 7.6 (Flowjo LLC) was used to analyze the data.

### Colony Formation Assay

Colony formation assays were performed as published previously with minor modifications (Bross et al., 2004). In brief, GIST48B, GIST54, and GIST226 cells were plated at 5000 cells/well in 6-well plates, respectively, and cultured in IMDM, for 14 days before treatment with bortezomib. After treatment with inhibitor for 7 days, the medium was removed, the cells were washed with PBS, and the cells were then stained with 0.5% crystal violet in methanol for 5 min. Excess stain was removed by washing with distilled water. Colonies were dissolved by using 900 µL glacial acetic acid and A_570_ was measured after colonies were photographed. The experiments were performed in duplicate wells and repeated three times.

### Cell Cycle Analysis

GIST48B, GIST54 and GIST226 cells in 6-well plates were trypsinized and washed with Hanks Balanced Salt Solution at 72 h after treatment with bortezomib. Nuclear staining was with a DAPI-containing PI solution and the cell suspension was immediately analyzed in a flow cytometer (BD Accuri C6, BD Biosciences). Data analysis was performed using Flow Jo and CFlow Plus. The experiments were performed in duplicate wells and repeated three times.

### *In Vitro* Wound-Healing Assays

Wound-healing studies were carried out as described previously (Shaw et al., 2001). Briefly, slashes were created in near-confluent cell cultures using the tip of a P-100 pipetman after addition of bortezomib (1 nM and 5 nM). Plates were photographed at day 0, 1, and 3 using a Leica DMI 3000B inverted microscope (Leica Microsystems). Experiments were performed in triplicate.

### Cell Migration and Invasion

Migration and invasiveness of GIST cells were evaluated by the Matrigel assay (Collaborative Research Inc.), as described previously (Yang et al., 2007). Briefly, Matrigel was diluted with IMDM in a 1:2 ratio and then coated onto 24-well inserts (Boyden chamber) with a 12-μm pore size, and then incubated at 37°C overnight. GIST48B and GIST54 cells (4 × 10^4^) were treated with bortezomib, followed by suspension in 0.5 ml of 0.5% serum-containing IMDM and seeded on the top chamber of each well with 1.5 ml of 15% serum-containing medium added to the bottom chamber, the higher serum content in the bottom chamber providing a chemotactic gradient. After 48 h, noninvasive cells that remained on the top surface of the filter were removed using a cotton swab and cells that remained adherent to the underside of the membrane were fixed in 4% formaldehyde and stained with 0.1% Crystal violet. Invasive cells were dissolved by using 900 µL glacial acetic acid and A_570_ was measured after these cells were photographed. Experiments were performed in triplicate.

### Statistical Analysis

Student’s t-tests were performed on data from cells treated with control DMSO or bortezomib. Statistically significant differences between untreated control and inhibitor treatment were defined as *p < 0.05, **p < 0.01, ***p < 0.001, and ****p < 0.0001.

## Results

### The Initial Inhibitor Screening in KIT Independent Gastrointestinal Stromal Tumor

**FIGURE 1:**
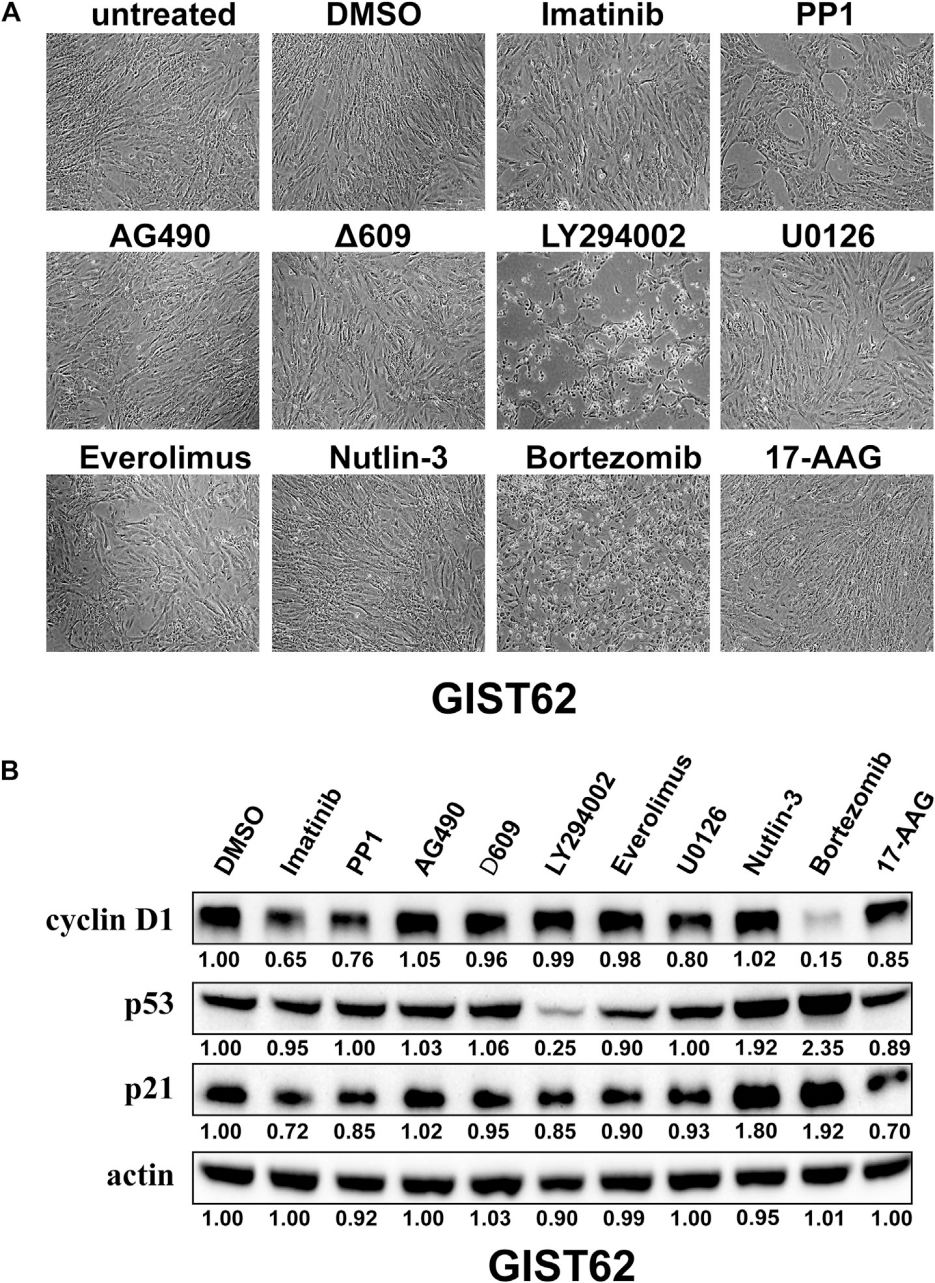
**(A)** Cell growth was evaluated in KIT-independent GIST cell line **(**GIST62) after treatment with Imatinib (1 μM), PP1 (1 μM), AG490 (10 μM), Δ609 (1 μM), LY294002 (50 μM), U0126 (10 μM), Everolimus (1 μM), Nutlin-3 (5 μM), Bortezomib (100 nM), and 17-AAG (0.5 μM) for 72 h. **(B)** Immunoblotting evaluated expression of cyclin D1, p53, and p21 in GIST62 cells after treatment with the bortezomib for 72 h. Actin stain is a loading control. Linear capture quantitation of immunoblotting chemiluminescence signals, using an ImageQuant LAS4000. Intensity values are standardized to the DMSO control.

Signaling pathways that regulate KIT independent GIST proliferation and/or survival were evaluated by drug inhibitor treatments in KIT independent GIST cell line (GIST62). As shown in Table 1, these initial drug screening studies used inhibitors of PDGFR and KIT (imatinib), Src-family kinases (PP1), JAK (AG490), PLCγ (∆609), PI3-K (LY294002), mTOR (Everolimus), MEK (U0126), Nutlin-3 (MDM2), proteasome inhibition (bortezomib), and HSP90 (17-AAG). The cell images and immunoblotting were evaluated in KIT-independent GIST62 after treatment with the different inhibitors for 72 h (Figure 1). The most dramatic anti-proliferative or pro-apoptotic effects were observed after PI3-K inhibition and proteasome inhibition, but not in other signaling suppression, as compared to DMSO control (Figure 1A). Immunoblotting evaluation showed that the greatest inhibition of cyclin D1 expression was obtained with bortezomib. Mild inhibition was seen after treatment with imatinib, PP1, and U0126, and little effects were observed after treatment with other inhibitors. Bortezomib and Nutlin-3 treatment resulted in the increase of p53 and p21 expression whereas other inhibitors had little effect on p53 and p21 expression (Figure 1B).

**TABLE1:** Screening of small molecular inhibitors in KIT-independent GIST62.

Targets	Inhibitors	Concentrations (μM)
KIT	Imatinib	1.0
SRC	PP1	5.0
JAK	AG490	10
PLCγ	△609	1.0
PI3K	LY294002	50
MEK	U0126	1.0
MTOR	Everolimus	1.0
MDM2	Nutlin-3	5.0
26S Proteasome	Bortezomib	0.1
HSP90	17-AAG	0.5

### Bortezomib Treatment Downregulates Cyclin D1 and YAP/TAZ in KIT-Independent Gastrointestinal Stromal Tumor

**FIGURE 2:**
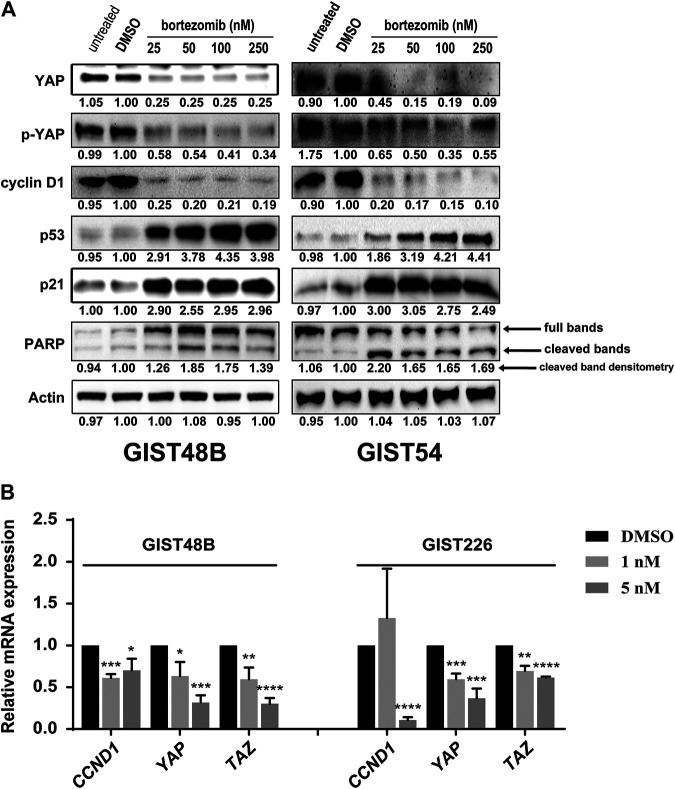
**(A)** Immunoblotting evaluated expression of cyclin D1, YAP, p-YAP, p53, p21, and pro-apoptotic marker PARP in KIT-independent GIST cell lines (GIST48B and GIST54) after treatment with bortezomib for 72 h. Actin stain is a loading control. Linear capture quantitation of immunoblotting chemiluminescence signals, using an ImageQuant LAS4000. Intensity values are standardized to the DMSO control. **(B)** Quantitative RT-PCR evaluations of *CCND1*, *YAP*, and *TAZ* transcript expression in GIST48B and GIST226 after treatment with bortezomib for 24 h. Data were normalized to DMSO control and represent the mean values (±s.d.) from triplicate assays, and were averaged from two independent experiments. Statistically significant differences between DMSO control and bortezomib treatment are presented as *p < 0.05, **p < 0.01, ***p < 0.001, ****p < 0.0001.

Our recent study showed that cyclin D1 expression and Hippo/YAP activation were significantly increased in KIT independent GIST cells, and inhibition of cyclin D1 and YAP resulted in anti-proliferative and pro-apoptotic effects (Ou et al., 2019). In addition, the initial study showed that bortezomib treatment induced the degradation of cyclin D1 and expression of p53 and p21 in GIST62 (Figure 1B). Thus, we next investigated the effects of bortezomib on cyclin D1 and YAP expression in other three KIT independent GIST cell lines (GIST48B, GIST54, and GIST226). Immunoblotting evaluation was performed after treatment with bortezomib for 48 or 72 h. Bortezomib treatment resulted in inhibition of YAP, p-YAP, and cyclin D1 expression, induction of cell cycle checkpoint p53 and p21, and PARP cleavage by pro-apoptotic protein maker in GIST48B, GIST54, and GIST226 (Figure 2A and Supplementary Figures S1, S2). Signaling protein expression quantitations are shown in Figure 2A and Supplementary Figures S1, S2. We further investigated expression levels of *CCND1*, *YAP*, and *TAZ* mRNA by qRT-PCR in KIT-independent GIST cell lines (GIST48B and GIST226) after treatment with bortezomib for 24 h. Bortezomib treatment inhibited *CCDN1*, *YAP*, and *TAZ* transcripts in these GIST cell lines (Figure 2B), which was consistent with their protein expression (Figure 2A and Supplementary Figures S1, S2).

### Targeting of Cyclin D1 and YAP by Bortezomib Inhibits Cell Growth and Induces Apoptosis in KIT Independent Gastrointestinal Stromal Tumor

**FIGURE 3:**
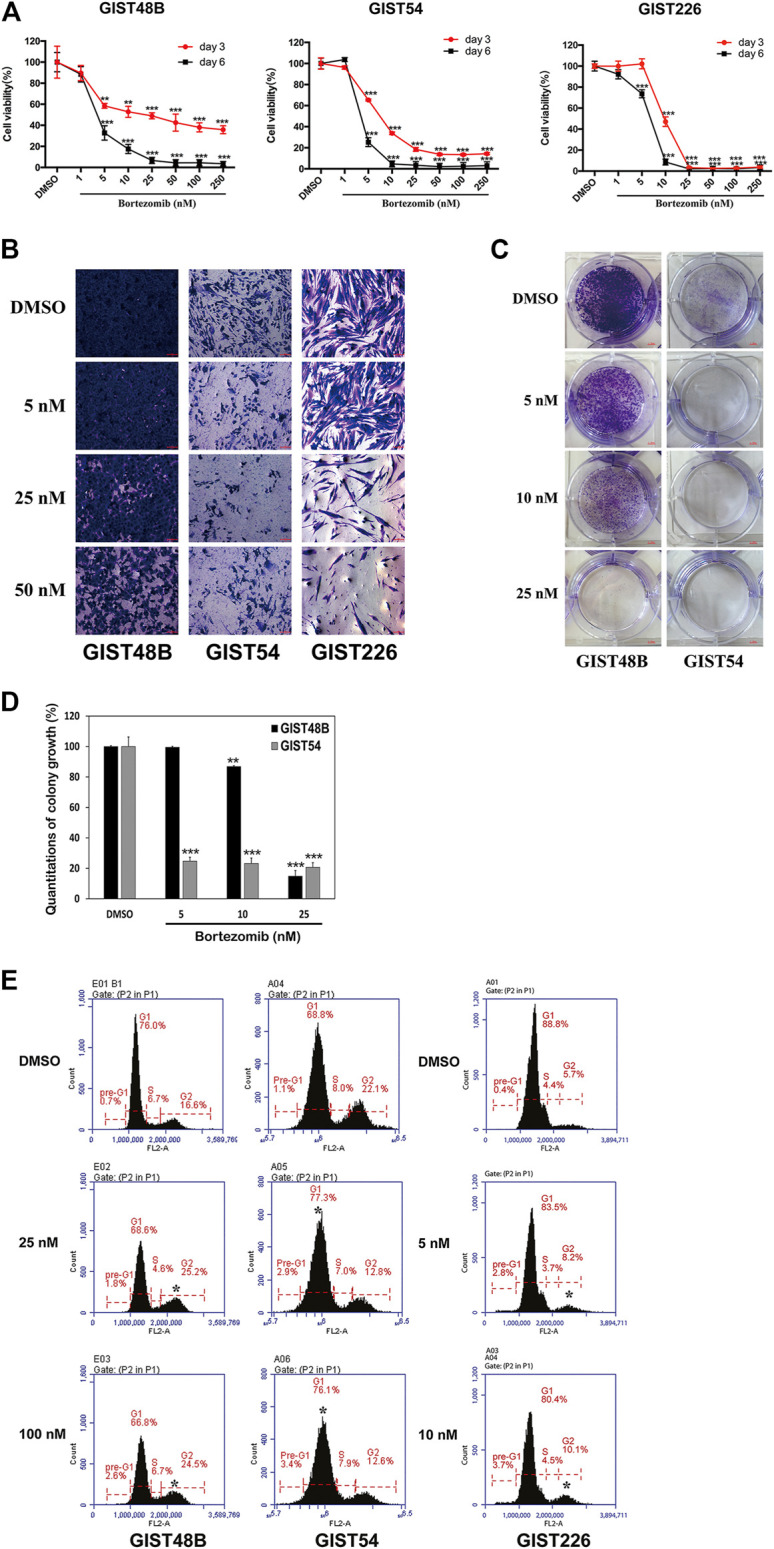
Anti-proliferative effects of bortezomib in KIT-independent GIST cell lines (GIST48B, GIST54, and GIST226) were assessed by cell viability, cell imaging, colony formation assays, and cell cycle analysis. **(A)** Cell viability was evaluated by MTT assays in GIST48B, GIST54, and GIST226 cell lines, 3 and 6 days after treatment with bortezomib (1, 5, 10, 25, 50, 100, and 250 nM). Data were normalized to DMSO control and represent the mean values (±s.d.) from quadruplicate cultures, and were averaged from two independent experiments. Statistically significant differences between DMSO control and bortezomib treatment are presented as **p < 0.01, ***p < 0.001. **(B)** Cell culture appearance of GIST48B, GIST54, and GIST226, when evaluated at 72, 48, and 48 h after treatment with bortezomib (5, 25, and 50 nM) and staining with crystal violet, showing dramatic growth inhibition compared to DMSO-treated control cultures. Scale bars: 100 μm. **(C)** Colony growth assays were performed at 7 days after treatment with bortezomib (5, 10, and 25 nM). Colony growth experiments were performed in triplicate. Bortezomib treatment led to a greater reduction in colony formation and size in GIST48B and GIST54 than the DMSO control. **(D)** Quantitation (A_570_) of GIST48B and GIST54 cell colony growth after treatment with bortezomib for 7 days. Statistically significant differences between untreated control and bortezomib treatments are presented as **p < 0.01, ***p < 0.001. **(E)** Cell cycle analysis was performed 72 h after treatment with bortezomib (25 and 100 nM). GIST48B, GIST54, and GIST226 showed a cell population increase in G1/G2-phase, accompanied by a decrease in G2/G1-and S-phase. Cell cycle experiments were performed in triplicate. Statistically significant differences between DMSO control and bortezomib treatment are presented as *p < 0.05.

Cell proliferation, as assessed using MTT assay, was strongly inhibited in all KIT independent cell lines after proteasome inhibition by bortezomib. Cell proliferation IC50s at Day 6 were 3.5 nM for GIST48B, 3 nM for GIST54, and 6.5 nM for GIST226, suggesting that bortezomib anti-proliferative effects are more pronounced in KIT-independent GIST cells (Figure 3A). Cell growth of GIST48B, GIST54, and GIST226 was reduced dramatically, when evaluated with crystal violet staining at 72 h after treatment with bortezomib (Figure 3B). Colonies of GIST cells treated with bortezomib (GIST48B, GIST54, and GIST226) were fewer and smaller in size when compared with DMSO-treated cells (Figure 3C and Supplementary Figures S3, S4). Relative to DMSO, colony formation decreased by 85% (GIST48B), 75% (GIST54), and 85% (GIST226) in cells treated with 25 nM, 5 nM, or 2.5 nM bortezomib, respectively (Figure 3D and Supplementary Figure S4). GIST48B, GIST54, and GIST226 cells were further evaluated by cell cycle assays (Figure 3E). Bortezomib treatment in GIST48B and GIST226 resulted in an increase of G2 peaks, from 16.6% and 5.7% in the DMSO-treated cells to 25.2% and 8.2% in bortezomib (25 nM) -treated cells, respectively. Bortezomib treatment in GIST54 resulted in an increase of G1 peaks, from 68.8% in the DMSO-treated cells to 77.3% in bortezomib (25 nM) -treated cells (Figure 3E and Table 2).

**TABLE 2:** Cell cycle analyses (%), as shown in Figure 3E, after bortezomib treatment.

Bortezomib	GIST48B					GIST54					Bortezomib	GIST226				
(nM)	G_1_	G_2_	S	Pre-G11		G_1_	G_2_	S	Pre-G1		(nM)	G_1_	G_2_	S	Pre-G1	
DMSO	76.0	16.6	6.7	0.7		68.8	22.1	8.0	1.1		DMSO	88.8	5.7	4.4	0.4	
25	68.6	25.2	4.6	1.8		77.3	12.8	7.0	2.9		5	83.5	8.2	3.7	2.8	
100	66.8	24.5	6.7	2.6		76.1	12.6	7.9	3.4		10	80.4	10.1	4.5	3.7	

**FIGURE 4:**
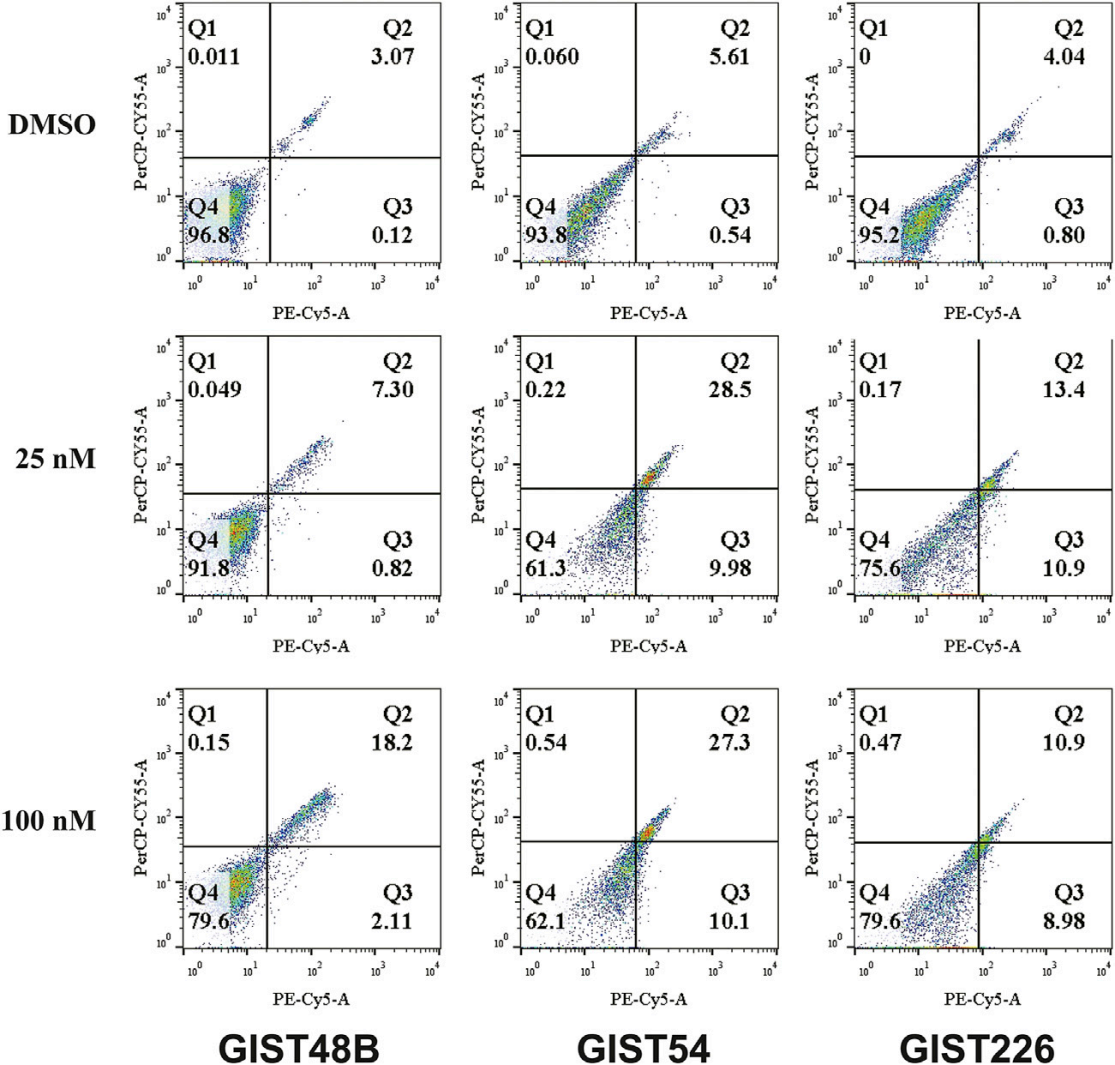
Apoptosis assays following bortezomib (25 and 100 nM) treatment for 72 h were performed with the PE Annexin V Apoptosis Detection Kit I. Apoptosis experiments were performed in triplicate.

In apoptotic assays, treatment of GIST48B, GIST54, and GIST226 with bortezomib for 72 h showed a greater increase in apoptotic cells, as compared with DMSO control (bortezomib 25 nM: 8.17% for GIST48B; 38.7% for GIST54; and 24.47% for GIST226; 100 nM: 20.31% for GIST48B; 37.4% for GIST54; and 19.88% for GIST226) (Figure 4 and Table 3).

**TABLE 3:** Cell apoptosis analyses (%), as shown in Figure 4, after bortezomib treatment.

Bortezomib	GIST48B						GIST54							GIST226			
(nM)	Q_1_	Q_2_	Q_3_	Q_4_		Q1		Q_2_	Q_3_	Q_4_			Q1		Q_2_	Q_3_	Q_4_
DMSO	0.011	3.07	0.12	96.8		0.06		5.61	0.54	93.8			0		4.04	0.80	95.2
25	0.049	7.30	0.82	91.8		0.22		28.5	9.98	61.3			0.17		13.4	10.9	75.6
100	0.15	18.2	2.11	79.6		0.54		27.3	10.1	62.1			0.47		10.9	8.98	79.6

### Targeting of Cyclin D1 and YAP by Bortezomib Inhibits Migration and Invasiveness in KIT Independent Gastrointestinal Stromal Tumor

**FIGURE 5:**
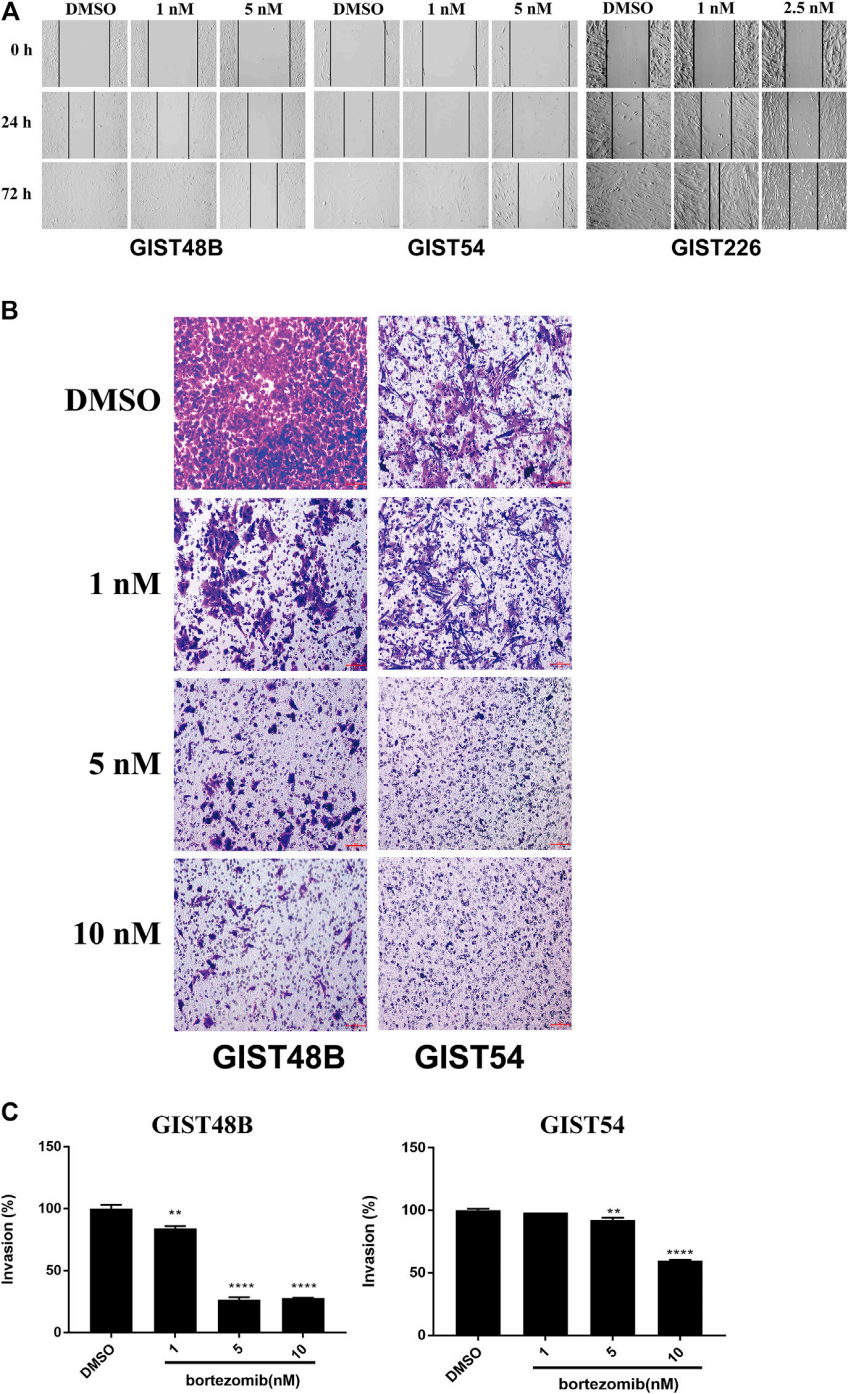
*In vitro* wounding assays **(A)** and transwell migration assays **(B)** show that bortezomib treatment effectively inhibits migration and invasion of KIT-independent GIST cell lines (GIST48B, GIST54, and GIST226). Scale bars: 100 μm. Wound healing and transwell experiments were performed in triplicate. **(C)** Quantitation (A_570_) of KIT-independent GIST cell (GIST48B and GIST54) invasiveness after treatment bortezomib for 72 h. Statistically significant differences between untreated control and bortezomib treatments are presented as **p < 0.01, ****p < 0.0001.

Wound-healing assays in cyclin D1 and YAP -overexpressed GIST48B, GIST54, and GIST226 cells demonstrated that downregulation of cyclin D1 and YAP by bortezomib resulted in marked inhibition of wound closure at 24–72 h, whereas the wounds were healed in all cell lines after 72 h of DMSO exposure (Figure 5A). Similarly, matrigel assays demonstrated 75% and 45% inhibition of invasiveness after bortezomib treatment (10 nM) in GIST48B and GIST54, respectively, compared with the DMSO control (Figures 5B,C).

## Discussion

One more challenging imatinib or other small molecular RTK inhibitor-resistant mechanism is loss of the oncogenic KIT protein expression (Fletcher et al., 2003; Debiec-Rychter et al., 2005). In our recent study, we identified distinctive biologic features in KIT-independent, imatinib-resistant GISTs (Tu et al., 2018; Ou et al., 2019). Overexpression of cyclin D1 and JUN, and activation of Hippo/YAP signaling were observed in KIT independent GISTs, but not in KIT dependent GISTs. Knockdown of cyclin D1, JUN, or YAP/TAZ showed dramatic anti-proliferative effects in KIT independent GISTs. Further studies demonstrated that PRKCQ, JUN, and the Hippo pathway coordinately regulated GIST cyclin D1 expression (Ou et al., 2019). Thus, we propose that targeting these pathways could be effective therapeutically for these untreatable tumors.

In the current report, we initially evaluated which small molecular inhibitors suppressed cyclin D1 expression in KIT-independent GIST. These drugs included imatinib (PDGFR and KIT), PP1 (Src-family kinases, SFKs), AG490 (JAK), ∆609 (PLCγ), LY294002 (pan-PI3-K), Everolimus (mTOR), U0126 (MEK), Nutlin-3 (MDM2), bortezomib (proteasome inhibition), and 17-AAG (HSP90). This was accomplished by targeting various pathways implicated in GIST proliferation, survival, or drug resistance. The SFKs have been found to be a potential therapeutic target in GIST given that PP2 or SU6656, which suppresses SKF inhibits GIST cell line proliferation, and activation of SFK on the Golgi is essential for oncogenic KIT signaling (Obata et al., 2017). Similarly, PI3-K inhibition by LY294002 resulted in substantial apoptosis and markedly decreased cell proliferation in the imatinib-sensitive and -resistant GISTs. MEK and mTOR inhibition by U0126 and RAD001 (Everolimus) showed moderate anti-proliferative effects in GIST cells, respectively (Bauer et al., 2007). These observations were also confirmed in KIT-independent GIST cells (Figure 1). The previous studies have shown that PI3K/AKT signaling inhibition by GDC0941 or shRNAs induced p53 expression in mesothelioma via inactivation of MDM2 (Zhou et al., 2014). However, p53 protein expression was reduced in GIST62 after treatment with LY294002 (50 µM) for 72 h (Figure 1B), which was in line with another early report that LY294002 (50 µM) treatment induced the maximal expression of p53 mRNA and protein in gastric cancer cell line SGC7901 at 6 hours and 24 h, respectively, but expression of p53 mRNA and protein was decreased after that time (Xing et al., 2008). This may be one reason that LY294002 is a broad-spectrum inhibitor targeting PI3-K (Chaussade et al., 2007). CK2 (Gharbi et al., 2007), and DNA-PK (Davidson et al., 2013), specifically in high-dose condition. Although the current data is compelling showing distinct antitumoral effects with LY294002 (Figure 1A), cyclin D1 expression was not suppressed after PI3-K inhibition (Figure 1B), which has been found to play crucial oncogenic role in KIT-independent GIST (Ou et al., 2019). Further studies will address the anti-tumor effects and mechanisms of LY294002 treatment in this challenging subset of GISTs. Also, it has been found that stem cell factor (SCF) mediated activation of wild-type KIT phosphorylates PLCγ (Linnekin, 1999), and actives JAK/STATs signaling pathway in GISTs (Duensing et al., 2004). The subsequent study shows that MDM2 inhibitor Nutlin-3 induces p53 expression and leads to moderate anti-proliferative effects in p53 wildtype GIST cells (Henze et al., 2012). The current report shows that Nutlin-3 treatment also mildly inhibited KIT-independent GIST62 cell growth through induction of p53 and p21 (Figure 1). Bortezomib treatment resulted in apoptosis in GIST cells through H2AX upregulation and degradation of KIT protein expression (Bauer et al., 2010). Additionally, targeting of ubiquitin-proteasome by second generation inhibitors of the 20S proteasome (delanzomib, carfilzomib and ixazomib) shows dramatic anti-proliferative effects in GISTs (Rausch et al., 2020). Furthermore, KIT oncoprotein expression is suppressed after treatment with a variety of HSP90 inhibitors, and results in anti-proliferative and pro-apoptotic effects (Bauer et al., 2006; Smyth et al., 2012).

In the KIT-independent GIST62, the screening of drugs demonstrated that bortezomib treatment resulted in anti-tumor effects through degradation of cyclin D1 and induction of checkpoint p53 and p21 (Figure 1), indicating that targeting of cyclin D1 by bortezomib inhibits KIT-independent GIST cell growth. Therefore, we next investigated the effects of bortezomib on expression of cyclin D1, p-YAP, and YAP proteins and transcripts (Figures 2A,B, and Supplementary Figures S1, S2), cell viability (Figure 3A), colony formation (Figures 3C,D, and Supplementary Figures S3, S4), cell cycle (Figure 3E and Table 2), apoptosis (Figure 4 and Table 3), migration (Figure 5A), and invasiveness (Figures 5B,C) in three KIT-independent GIST cell lines (GIST48B, GIST54, and GIST226). Bortezomib treatment showed significant anti-proliferative, pro-apoptotic and anti-metastatic effects in these cells via inhibition of cyclin D1 and YAP expression, and induction of tumor suppressor p53 and p21 (Figures 2–5). In our early study, activated Hippo signaling/YAP-TAZ positively regulated cyclin D1 protein overexpression in KIT-independent GIST cells (Ou et al., 2019), and in the current report bortezomib treatment co-targeted expression of YAP/TAZ and cyclin D1 transcripts and proteins.

In the present report, it seems that anti-proliferative and pro-apoptotic effects of bortezomib are not dose-dependent under the tested concentrations (25–250 nM). Possibly, 25 nM (even ∼10 nM) of bortezomib may reach the maximum dose for these effects. However, immunoblotting evaluation and qRT-PCR showed that expression of cyclin D1 and YAP was inhibited in a low bortezomib dose-dependent manner (below 10 nM) (Supplementary Figure S2 and Figure 2B), which was in line with anti-proliferative effects (Figure 3A). In fact, dose-dependent effects of bortezomib were also observed in analysis of cell growth (Figure 3B), colony formation (Figures 3C,D), migration (Figure 5A), and invasiveness (Figures 5B,C) in high dose-dependent manner. On the basis of cyclin D1 and YAP silencing levels and anti-proliferative effects of bortezomib below 10 nM, there is a reasonable prospect that these phenotypes are affected in bortezomib concentration dependent manner, especially in low dose bortezomib.

In addition, the dramatic anti-proliferative effects (Cell viability IC50s at day 6 were 3.5 nM for GIST48B, 3 nM for GIST54, and 6.5 nM for GIST226, respectively, via targeting cyclin D1 and YAP/TAZ expression in low dose of bortezomib (1–10 nM) (Supplementary Figure S2 and Figure 2B) have demonstrated that bortezomib effects are more pronounced against KIT-independent GIST cells in vitro-based study. This indicates that bortezomib may have a strong potential in this subset of GISTs *in vivo*. Rausch et al. also found that all three compounds (second-generation inhibitors of the 20S proteasome carfilzomib, ixazomib or delanzomib) were highly effective for GIST cell viability and apoptosis (IC50s as low as 9 nM) with a profile similar to bortezomib, and for tumor growth in KIT-dependent GIST cell line xenografts and patient-derived xenografts (Rausch et al., 2020). The previous report has also found that anti-proliferation IC50s of bortezomib were 2–11 nM in multiple myeloma (MM) and mantle cell lymphoma (MCL) cell lines, which is comparable to GISTs (Manni et al., 2013). According to oral dosing between 7.8 mg/kg and 13 mg/kg in myeloma models (Piva et al., 2008; Sanchez et al., 2010), delanzomib dose of 8 mg/kg have shown well translation into the human setting, such as tumor volume, weight, and general health of the mice (Van Looy et al., 2014; Rausch et al., 2020). Thus, we have reasons to speculate anti-tumoral effects of bortezomib *in vivo* in KIT-independent GISTs, indicating that bortezomib or delanzomib treatment is the rationale as one clinical strategy for this untreatable GISTs.

These pro-apoptotic and anti-proliferative findings constitutes early proof-of-principle. A potential novel therapeutic approach for the untreatable KIT-independent GISTs, which constitutes a minor but challenging portion of the resistance mechanisms that the majority of GISTs eventually develop against standard-of-care TKI treatment. More importantly, the anti-tumor effects of bortezomib targeting cyclin D1 and Hippo/YAP will need to be evaluated *in vivo* in KIT-independent GISTs.

In conclusion, bortezomib is a potential drug for induction of apoptosis and anti-proliferation in GIST cells with resistance to imatinib, including resistance via acquisition of secondary *KIT* mutations (Bauer et al., 2010; Rausch et al., 2020) and loss of oncogenic KIT proteins with cyclin D1 overexpression. This indicates that inhibition of cyclin D1 and Hippo/YAP signaling by proteasome inhibitor bortezomib might be a biologically rational therapeutic strategy for KIT-independent GIST.

## Data Availability

The original contributions presented in the study are included in the article/Supplementary Material, further inquiries can be directed to the corresponding author.
