# Supplementary material for: Proteasome Inhibition Suppresses KIT-Independent Gastrointestinal Stromal Tumors Via Targeting Hippo/YAP/Cyclin D1 Signaling
Source: Front Pharmacol. 2021 May 6;12:686874. doi: 10.3389/fphar.2021.686874 (PMC8134732; doi:10.3389/fphar.2021.686874)
Supplement: Supplementary file 1 [file DataSheet1.PDF]

SFigure 1

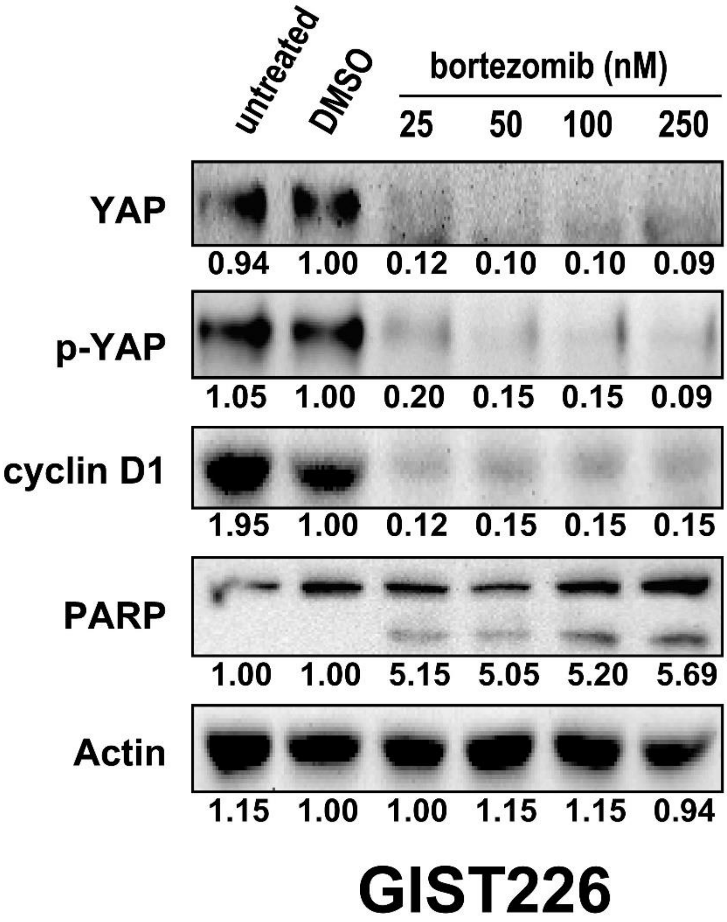

SFigure 1. Immunoblotting evaluated expression of cyclin D1, YAP, p-YAP, and pro-apoptotic marker PARP in KIT-independent GIST226 cells after treatment with bortezomib for 48 hours. Actin stain is a loading control. Linear capture quantitation of immunoblotting chemiluminescence signals, using an ImageQuant LAS4000. Intensity values are standardized to the DMSO control.

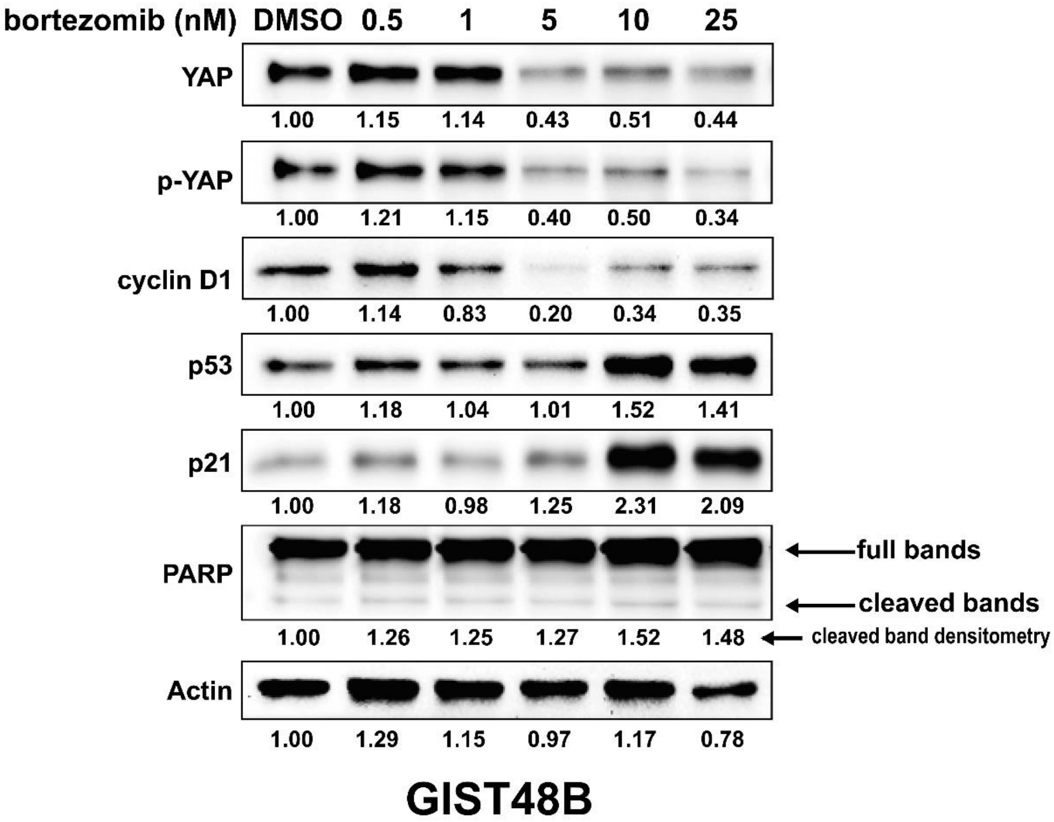

SFigure 2. Immunoblotting evaluated expression of cyclin D1, YAP, p-YAP, and pro-apoptotic marker PARP in KIT-independent GIST48B cells after treatment with bortezomib (0.5, 1, 5, 10, and 25 nM) for 72 hours. Actin stain is a loading control. Linear capture quantitation of immunoblotting chemiluminescence signals, using an ImageQuant LAS4000. Intensity values are standardized to the DMSO control.

SFigure 3

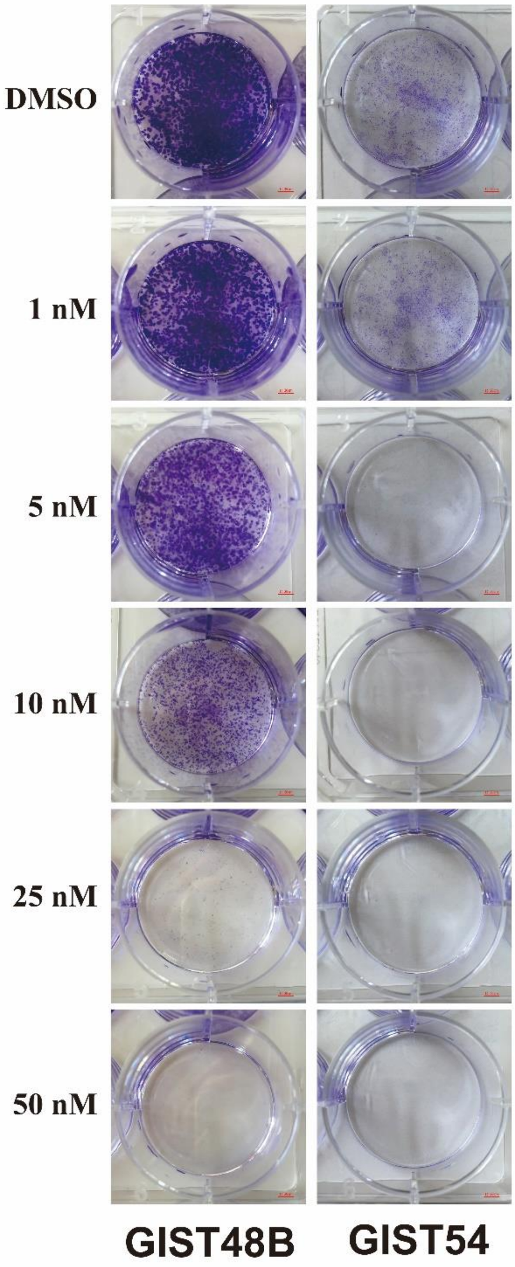

SFigure 3. Anti-proliferative effects of bortezomib in KIT-independent GIST cell lines (GIST48B and GIST54) were assessed by colony formation assays. Colony growth assays were performed at 7 days after treatment with bortezomib (1, 5, 10, 25. and 50 nM). Colony growth experiments were performed in triplicate. Bortezomib treatment led to a greater reduction in colony formation and size in GIST48B and GIST54 than the DMSO control.

SFigure 4

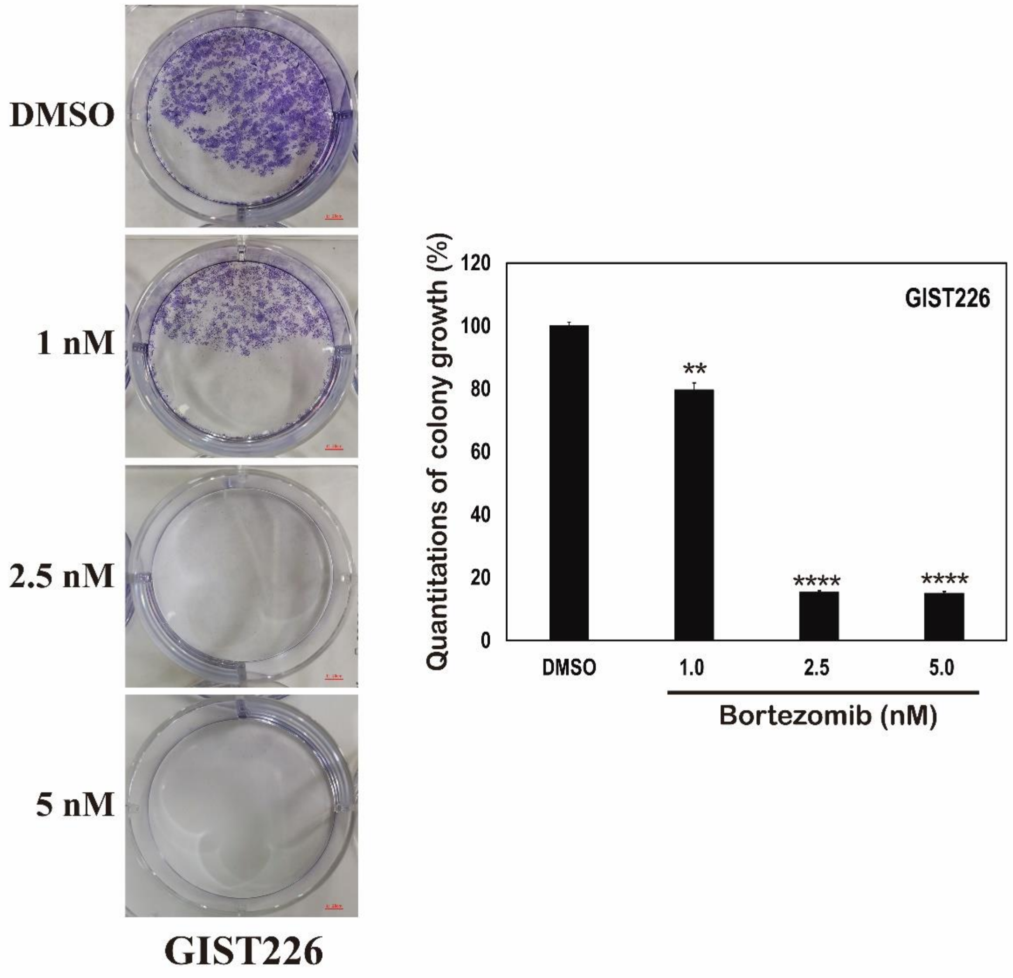

SFigure 4. Anti-proliferative effects of bortezomib in GIST226 were assessed by colony formation assays. **Left panel**: Colony growth assays were performed at 7 days after treatment with bortezomib (1.0, 2.5, and 5 nM). Colony growth experiments were performed in triplicate. Bortezomib treatment led to a greater reduction in colony formation than the DMSO control; **Right panel:** Quantitation (A_{570}) of GIST226 cell colony growth after treatment with bortezomib for 7 days. Statistically significant differences between untreated control and bortezomib treatments are presented as $^{**}p < 0.01$, $^{****}p < 0.0001$.
